# Explorative data analysis of MCL reveals gene expression networks implicated in survival and prognosis supported by explorative CGH analysis

**DOI:** 10.1186/1471-2407-8-106

**Published:** 2008-04-16

**Authors:** Steffen Blenk, Julia C Engelmann, Stefan Pinkert, Markus Weniger, Jörg Schultz, Andreas Rosenwald, Hans K Müller-Hermelink, Tobias Müller, Thomas Dandekar

**Affiliations:** 1 Department of Bioinformatics, University of Würzburg, Biozentrum, Am Hubland, D-97074 Würzburg, Germany; 2 Institute for Pathology, University of Würzburg, Josef-Schneider-Str. 2, D-97080 Würzburg, Germany

## Abstract

**Background:**

Mantle cell lymphoma (MCL) is an incurable B cell lymphoma and accounts for 6% of all non-Hodgkin's lymphomas. On the genetic level, MCL is characterized by the hallmark translocation t(11;14) that is present in most cases with few exceptions. Both gene expression and comparative genomic hybridization (CGH) data vary considerably between patients with implications for their prognosis.

**Methods:**

We compare patients over and below the median of survival. Exploratory principal component analysis of gene expression data showed that the second principal component correlates well with patient survival. Explorative analysis of CGH data shows the same correlation.

**Results:**

On chromosome 7 and 9 specific genes and bands are delineated which improve prognosis prediction independent of the previously described proliferation signature. We identify a compact survival predictor of seven genes for MCL patients. After extensive re-annotation using GEPAT, we established protein networks correlating with prognosis. Well known genes (CDC2, CCND1) and further proliferation markers (WEE1, CDC25, aurora kinases, BUB1, PCNA, E2F1) form a tight interaction network, but also non-proliferative genes (SOCS1, TUBA1B CEBPB) are shown to be associated with prognosis. Furthermore we show that aggressive MCL implicates a gene network shift to higher expressed genes in late cell cycle states and refine the set of non-proliferative genes implicated with bad prognosis in MCL.

**Conclusion:**

The results from explorative data analysis of gene expression and CGH data are complementary to each other. Including further tests such as Wilcoxon rank test we point both to proliferative and non-proliferative gene networks implicated in inferior prognosis of MCL and identify suitable markers both in gene expression and CGH data.

## Background

Mantle cell lymphomas (MCL) make up about 6% of all cases of non-Hodgkin's lymphomas. They occur at any age from the late 30s to old age, are more common in the over 50 years old population and three times more common in men than in women. Morphologically, MCL is characterized by a monomorphic lymphoid proliferation of cells that resemble centrocytes. MCL is associated with a poor prognosis and remains incurable with current chemotherapeutic approaches. Despite response rates of 50–70% with many regimens, the disease typically relapses and progresses after chemotherapy. The median survival time is approximately 3 years (range, 2–5 y); the 10-year survival rate is only 5–10%.

The characteristic translocation t(11;14) leads to overexpression of Cyclin D1 in the tumor cells which therefore comprises an excellent marker in the diagnostic setting [1]. The present study is an effort to improve molecular insights and markers of the disease [2-6] to improve the diagnosis and potential therapeutic strategies. We used gene expression data from 71 cyclin D1-positive patients and coupled these to data on their corresponding chromosomal aberrations (n = 71). We found molecular markers in addition to cyclin D1 and characteristic antigens (shared with blood cells from which the tumor may develop) CD5, CD20 and FMC7 with the aim to better delineate the regulatory network regulated differently in MCL.

Starting from the proliferation signature [6] we compare long and short living patients subgroups "s" (survivor, above median of survival) and "b" (bad prognosis, below median of survival). Exploratory analysis of gene expression and CGH data shows new genes differentiating both subgroups, proliferation associated genes and non-proliferative genes. For clinical application a seven gene predictor is derived from these gene markers, distinguishing patients with good or bad survival prognosis. A Wilcoxon rank sum test on CGH data identifies specific changes on chromosome 9 and 7.

## Methods

### Data and Materials

MCL gene expression data (n = 71) were obtained from cDNA arrays containing genes preferentially expressed in lymphoid cells or genes known or presumed to be part of cancer development or immune function ("Lymphochip" microarrays [7]; data have been deposited at NCBI's Gene Expression Omnibus data repository under GEO series accession number GSE10793. We give also the resulting gene expression ratios [see Additional file 5] and the prognosis assigned to patients [see Additional file 6]. The dataset is completed by comparative genomic hybridization (CGH) data for each patient (n = 71). The samples were collected from cyclin D1-positive patients of several hospitals in the "Lymphoma and Leukemia Molecular Profiling Project" (LLMPP) [6].

### Statistical analysis

Most of the statistical analyses were performed using the "Genome Expression Pathway Analysis Tool" (GEPAT). This is a web-based platform for annotation (allowing also extensive re-annotation of the data), analysis and visualization of microarray gene expression data [8] including genomic, proteomic and metabolic features.

The database performs the analyses applying Bioconductor [9], an open source software for the analysis and comprehension of genomic data, based on the R programming language [10].

For identification of differentially expressed genes, GEPAT uses the "limma" package which offers moderate t-statistics [11,12]. It fits linear models on the gene expression values of each gene with respect to the groups which are compared. After that empirical Bayes shrinkage of the standard errors is performed. Due to its robustness the method can be applied to experiments with a small number of samples. To correct for multiple testing it offers three options, we chose the method by Benjamini and Hochberg [13].

For identifying all protein-protein network interactions GEPAT uses the "Search Tool for the Retrieval of Interacting Genes/Proteins" (STRING) [14]. The STRING database comprises known and predicted protein-protein interactions. The interaction information arises from genomic context, experiments, other databases, coexpression and textmining.

For explorative correspondence analysis and principal component analysis, functions from the R package "Modern Applied Statistics with S" (MASS) was applied [15]. A constrained or canonical correspondence analysis (CCA) [16] was performed using the vegan package [17].

The Wilcoxon rank sum test [18], a non-parametric statistical test, was applied to the CGH data. It tests here each of the chosen bands against the null hypothesis that there is no statistically significant difference between our proposed two MCL patients "b" and "s". The R package "survival" is used to calculate all Cox regression hazard models [19,20]. It examines the correlation between the given measurements and the survival data. For the exploratory analysis of the CGH data as well as for the new predictor of MCL overall survival, we used the Wald test to determine the significance of the association between the model and the outcome.

## Results

### Exploratory analysis and lymphoma prognosis

**Figure 1:**
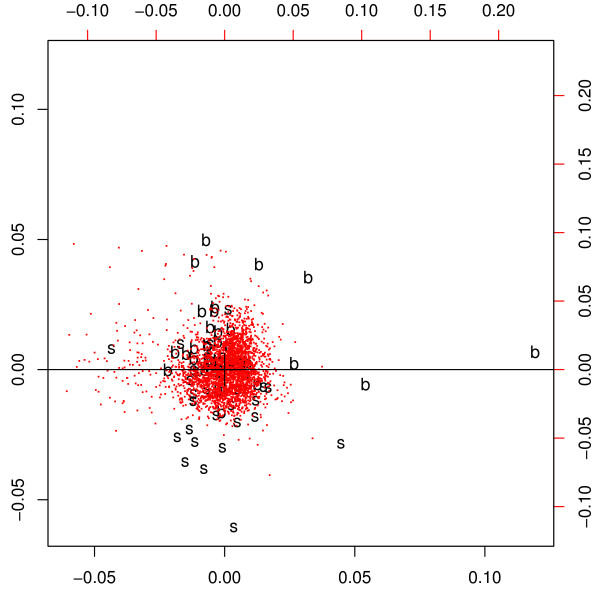
**Correspondence analysis identifies the two Mantle cell lymphoma subgroups**. The gene expression data are projected on the first two principal axes. The patients can be clearly separated by this exploratory analysis considering the 3.000 genes (red dots) of the highest variance. In the correspondence plot this is indicated by the horizontal separation line. The patients are labelled with "s" and "b" which represent the separation by the *median of the proliferation signature *into two different entities. Patients with a proliferation signature value smaller than the median are marked with ”s“ and the other patients with ”b“.

The survival time itself is the most obvious and biological meaningful parameter in which subgroups should show a big difference for realizing individual clinical treatment. We selected 3.000 genes with the highest variance and applied correspondence analysis (Figure 1). We found (71 MCL patients) that already the second axis separated almost perfectly the longer and the shorter living patients above and below the median of survival. Furthermore, this coincides well with the median of the proliferation signature [6] values in a multidimensional data space (see Methods). This finding was re-examined by exploratory data analysis of the genes of the proliferation signature and a huge amount of further genes. We ranked a total of 71 MCL patients according to their proliferation signature values and separated them according to the median. We define two groups – "s" for small and "b" for big proliferation signature with big difference in the survival time. Patients with a high proliferation signature value live shorter on average, than patients with a low proliferation signature value.

**Figure 2:**
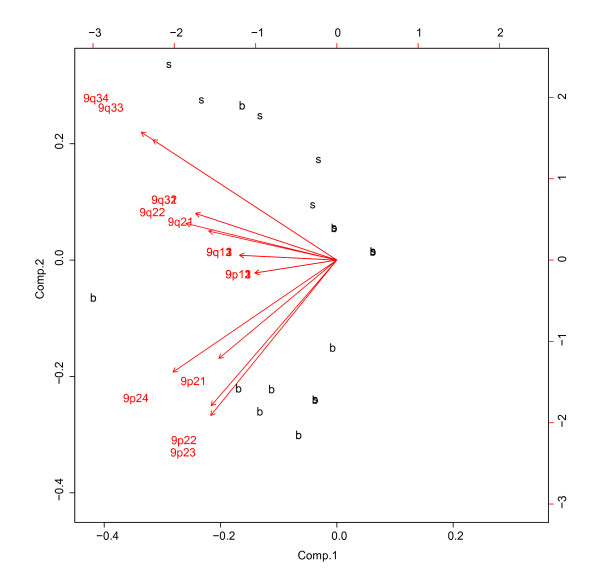
**Principal Component Analysis of chromosome 9 bands separating the "s" and "b" group**. The second principal component separates almost all patients of the subgroup "b" from the remain. They are grouped together close to the first four vectors, corresponding to the first four bands 9p24, 9p23, 9p22, 9p21, which go into the same direction and are of similar length. Remarkable are the vectors of the bands 9q33 and 9q34. They also are of similar length and go exactly into the same direction. Along their length, they congregate almost all patients of the type "s". This leads to the assumption, that the first four and the last two bands of chromosome 9 play a crucial role for "s" and "b" classification.

To each single chromosome of the CGH data exploratory data analysis was applied, correspondence analysis [see Additional file 1] and principal component analysis (Figure 2). Both methods are useful for exploring information and structures in data in order to get a first and unbiased impression. Principal components analysis reduces multidimensional data sets to lower dimensions for analysis. Correspondence analysis works similarly, but scales the data, such that both rows and columns can be visualized in one plot. Results show a strong correlation for four bands of chromosome 9, 9p24, 9p23, 9p22 and 9p21 and above median ("s") or below median patient survival ("b").

In the *correspondence analysis *plot [see Additional file 1], the four bands mentioned before attract most patients of the subgroup "b" and the 1st factor axis separates almost completely the two groups. Bands 9q33 and 9q34, are located relatively far away from the remaining ones. In Figure 2 the second principal component groups almost all the "b" – patients near the four bands 9p24, 9p23, 9p22, 9p21 with vectors of similar length and similar direction. The vectors of 9q33 and 9q34 include along their lengths almost all "s" samples. These results indicate that these six bands of chromosome 9 correlate with good and bad survival between patients. The principal component 1 is an interesting main component, carrying 51% of the variance, but non-trivial to link to a known phenotype (we investigated different possibilities including sex differences, cancer sub-types, patient accrual and correlation with different gene signatures).

Further exploratory data analysis was performed to merge the survival time and the CGH data by the Cox regression hazard model. A univariate Cox regression hazard model was performed on all available bands of the CGH data of all 71 patients. The mentioned four bands of chromosome 9 delivered amongst others the most significant results. The resulting bands are "9p24", "9p23", "9p22", "9p21", "9q31" and "9q32". These comprise the first four bands found on chromosome 9 by the analyses before.

### A compact predictor of survival with seven genes

Exploratory analysis pointed to differences between longer and shorter living MCL patients, but rather than forming two distinct subgroups, the patients constitute a coherent continuum. Therefore, the results of the exploratory analysis above were not additionally confirmed by classification tools. However, the differences in gene expression above and below the median of survival correlate well with different gene signatures identified before (proliferation signature) as well as with the new ones described in our study (non-proliferative signatures, see below). To improve survival predictions we further searched with univariate Cox regression hazard analysis for highly significant genes, which correlate strongly with the overall survival time. The cox regression was applied to all data points. However, the first 50 MCL samples served as training set for classification by gene signatures and the remaining data (21 patients) for validation. The idea was here to have a large training data set, but still keep a third of the available data for validation.

A four gene predictor with the genes CDC2, ASPM, tubulin-α and CENP-F reported in [6] could not be tested, as after reannotation by GEPAT [8], mapping of CENP-F seemed uncertain. Predictors with 4, 5 or 6 genes delivered not the same predictive power as the proliferation signature [6] (data not shown). The prediction power was calculated from the correct classification and misclassification for patients over or below the median of survival for 69 patients (the two patients with the median value were excluded).

However, we identified a set of seven genes delivering similar good prognosis separation. It includes (i) the well known key cell cycle kinase CDC2 [21,22], (ii) the "cell division cycle 20 homolog" (CDC20) required for anaphase and chromosome separation [23] and (iii) the salvage pathway gene HPRT1 (hypoxanthine phosphoribosyltransferase 1), three genes from the 20 genes proliferation signature of Rosenwald [6]. We get improved prediction power including four additional genes (Table 1): (i) centromere protein E (CENPE), a kinesin-like motor protein; it accumulates during G2 phase of cell cycle for chromosome movement or spindle elongation [24]. (ii) BIRC5 (baculoviral IAP repeat-containing 5 gene), an inhibitor of apoptosis (IAP gene family) is expressed in most tumours and in lymphoma [25], participates in the spindle checkpoint and associates with AURKB [26]. (iii) ASPM (abnormal spindle homolog) is essential for normal mitotic spindle function [27]. (iv) Insulin-like growth factor 2 mRNA binding protein 3 (IGF2BP3), is found in the nucleolus, is over-expressed in human tumours and represses IGF2 during late development [28-30].

**Table 1:** The genes of the survival predictor

**Acc**	**Gene**	**EnsemblID**	**Official full name**
6558	CENPE	ENSG00000138778	Centromeric protein E
7495	CDC20	ENSG00000117399	Cell division cycle protein 20 homolog
7892	HPRT1	ENSG00000165704	Hypoxanthine-guanine phosphoribosyltransferase
7019	CDC2	ENSG00000170312	Cell division control protein 2 homolog
7376	BIRC5	ENSG00000089685	Baculoviral IAP repeat-containing protein 5
6422	ASPM	ENSG00000066279	Abnormal spindle-like microcephaly-associated protein
5923	IGF2BP3	ENSG00000136231	IGF-II mRNA-binding protein 3

Univariate Cox regression hazard analysis revealed these seven genes best correlating with the survival time (see Material and Methods). The first column indicates the gene accession number in the data set (Acc), the second the gene name, followed by the Ensembl identifier and the official full name. The genes are ordered by their significance in decreasing order. CENPE is the most significant gene.

**Figure 3:**
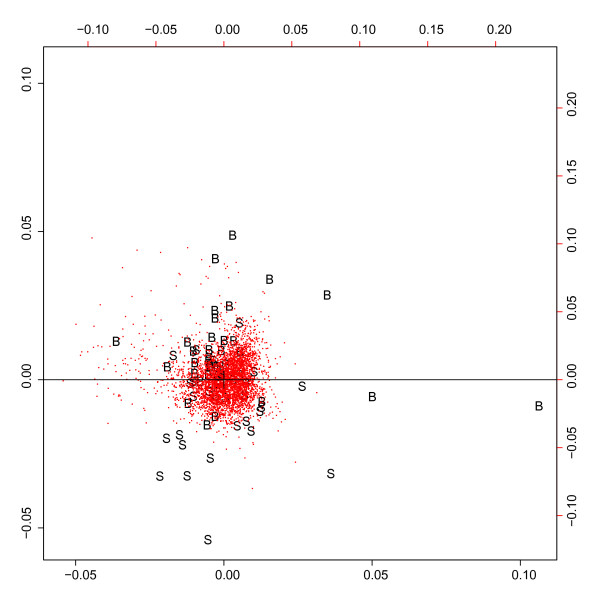
**Correspondence analysis separates two MCL subgroups derived by the 7 genes survival predictor**. The 3.000 genes with highest variance (red dots) separate between the two subgroups, which were delivered by the seven gene predictor and are drawn as "s" and "b". They were separated by the median of the predictor values. In contrast to the proliferation signature based predictor (Figure 1), the patients here show a little more overlap, but cluster clearly.

**Figure 4:**
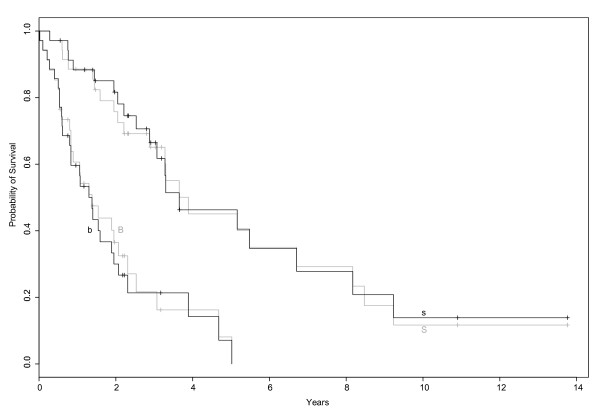
**Kaplan Meier plot of survival data in MCL subgroups**. The x-axis denotes the course of time in years and the y-axis marks the probability of survival. Both, the proposed proliferation signature (black) and the seven genes predictor (grey) separate clearly two risk groups in the survival data. The overlap between the patients of the two classifications is relatively high.

The seven genes were used to calculate a multivariate Cox regression hazard model and with its coefficients, a gene expression based survival estimator separated all 71 patients into two subgroups. Two patients had exactly the median of survival and were excluded in this comparison, 56 agreed with the classification according to the gene signatures, 13 did not. Compared to the proliferation [6] signature's ability to distinguish patients with good and bad survival prognosis (Figure 3), the seven gene predictor does it similarly well (Figure 4). The correlation between this classification and the "s" and "b" groups of the proliferation signature is overall about 0.62 and in our validation set (patients 51 – 71) it is 0.81.

A correspondence analysis of the 3.000 genes with the highest variance showed clear clustering of patients with good or bad prognosis, respectively (Figure 1). Using proliferation signature [6] (Figure 3), samples show a little overlap, but are again separated clearly.

Taken together, these results show that the seven gene predictor is able to distinguish patient prognosis as well as the complete proliferation signature, but with less effort.

### Protein networks and interactions differently regulated in good and bad prognosis tumors

**Figure 5:**
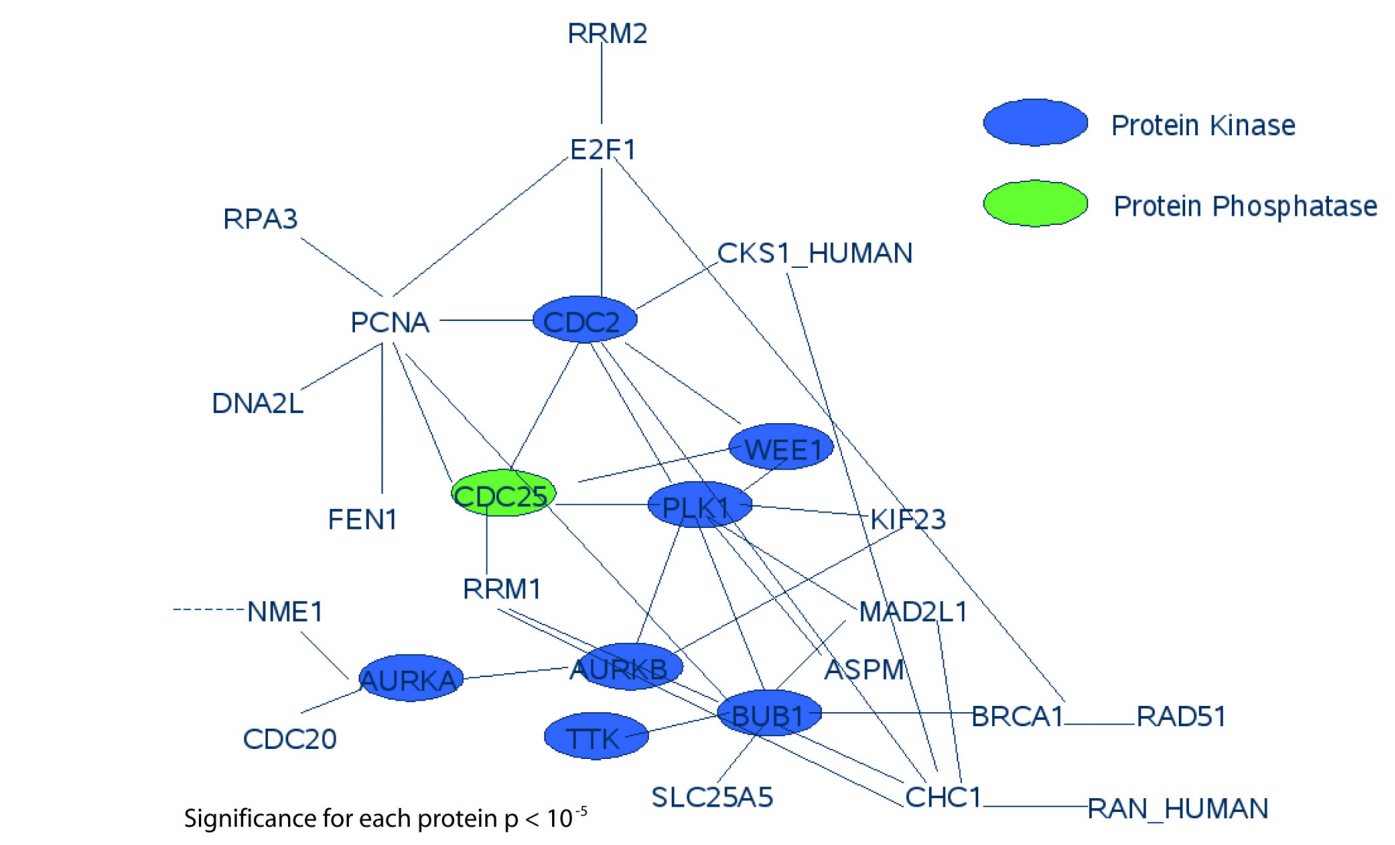
**Protein interaction network of significantly different expressed genes**. The genes encoding these proteins show a significant expression difference between the "s" and "b" group (moderate t-test). Remarkably CDC2 is involved in a small interaction network of protein kinases and almost all of these interaction partners (CDC25, WEE1, AURKB, AURKA, BUB1) are associated with the cell cycle.

**Figure 6:**
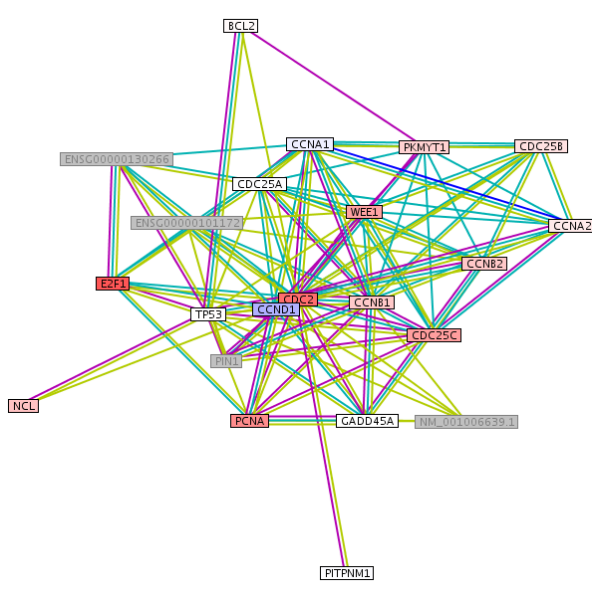
**Differences in gene expression of interaction partners of CDC2 in MCL subgroups**. In this network figure, red indicates high expression and blue low expression in the subgroup "b" of the proliferation signature. White indicates no gene expression difference and grey the unavailability of the gene in our data set. "Cell division cycle 2" (CDC2) gene interacts in different manners with "cyclin D1" (CCND1), "cell division cycle 25C"(CDC25C), "proliferating cell nuclear antigen"(PCNA), "E2F transcription factor 1"(E2F1) and WEE1. CDC2 and CCND1 are both required for the G1/S transition. The genes WEE1 and CDC25C phosphorylate and dephosphorylate the complexes bound with CDC2 in a cell cycle regulating manner. The "proliferating cell nuclear antigen" (PCNA) is involved in DNA replication whereas "E2F transcription factor 1" (E2F1) controls cell cycle and mediates cell proliferation and apoptosis. A cell cycle regulated transcription activator "Nucleolin" (NCL) shows little difference.

We found a dense regulatory network of interacting genes correlated with prognosis. Applying a moderate t-test, the well known cell division cycle 2 gene (CDC2/CDK1) for G1 to S and G2 to M transition [31,32] shows the most significant difference between the longer living "s" and the shorter living "b" patients (Table 2). Furthermore, its interaction partners according to HPRD database [33] show a significant up or down regulation comparing good and bad surviving patients (Figure 5), e.g. WEE1 and CDC25. Moreover, aurora kinases A, B [34] and BUB1 kinase activating the spindle checkpoint [35], are differently regulated between shorter and longer living patients. However, there are further genes involved in this network of directly interacting genes differently regulated in good or bad prognosis patients (Figure 5; Figure 6) such as (i) "proliferating cell nuclear antigen" (PCNA), a cofactor of DNA polymerase delta, helps to increase the processivity of leading strand synthesis during DNA replication in group "b". Because of its ability to interact with multiple partners, it is involved in Okazaki fragment processing, DNA repair, translation, DNA synthesis, DNA methylation, chromatin remodelling and cell cycle regulation [36]. (ii) E2F transcription factor 1 (E2F1), this protein can mediate both cell proliferation and p53-dependent/independent apoptosis [37]. It is lower expressed in group "s". (iii) Nucleolin is an abundant multifunctional phosphoprotein of proliferating and cancerous cells [38-41] and highly expressed in "b".

**Table 2:** Most significant genes separating good (s) and bad (b) prognosis

**Acc**	**Gene**	**Fold change**	**p-value**	**EnsemblID**
7019	CDC2	1.3737029	1.8651454E-13	ENSG00000170312
6632	NP_057427.3	0.94384	3.4574367E-13	ENSG00000117724
3399	UHRF1	1.1446086	1.5513529E-12	ENSG00000034063
5112	NP_060880.2	1.0916529	1.5513529E-12	ENSG00000123485
6994	AURKB	1.4594886	1.5513529E-12	ENSG00000178999
6388	MKI67	1.5062114	1.7304206E-12	ENSG00000148773
6721	Q9Y645_HUMAN	1.2185314	3.2408542E-12	ENSG00000140451
7024	BUB1	1.2488679	3.2408542E-12	ENSG00000169679
6392	NP_057427.3	1.3208085	3.2902188E-12	ENSG00000117724
5726	MKI67	1.4871315	3.6012686E-12	ENSG00000148773
6029	NP_057427.3	1.2980943	5.249176E-12	ENSG00000117724
7423	BIRC5	1.3726515	6.49239E-12	ENSG00000089685
4985	ASPM	1.3310171	7.281489E-12	ENSG00000066279
5754	KIF23	1.2461857	1.6424877E-11	ENSG00000137807
5271	ASPM	1.3205649	2.2259293E-11	ENSG00000066279
6104	KIF23	1.1683029	2.4981522E-11	ENSG00000137807

The most significant differentially expressed genes regarding good ("s") or bad ("b") prognosis determined with a moderate t-test. P-values were corrected for multiple testing [13]. The gene "cell division cycle 2" (CDC2), which is important for the transition G1 to S and G2 to M shows the biggest difference in gene expression between the two groups. This indicates that these cell cycle transitions are part of the difference between the two groups.

**Figure 7:**
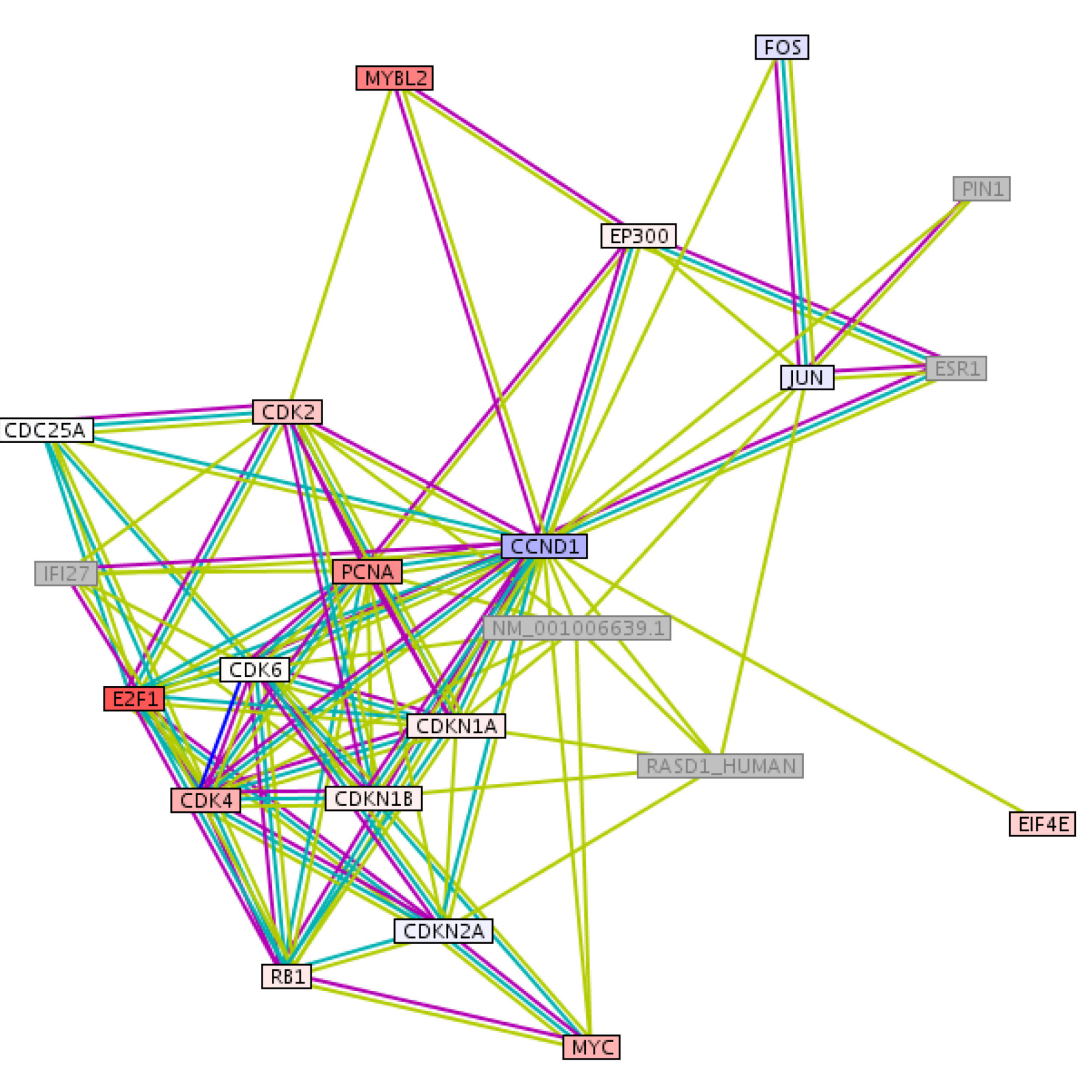
**Protein interaction partners of CCND1: Different gene expression in MCL subgroups**. The colors red, blue and grey mean "over expressed", "down regulated" (in "b") and "not available in the data set". FOS encodes for a leucine zipper protein and plays a role in regulation of cell proliferation, differentiation, transformation and tumorigenesis [58]. The JUN protein interacts directly with specific target DNA sequences to regulate gene expression [59] and is involved in tumorigenesis by cooperating with oncogenic alleles of Ras, an activator of the mitogen activated protein kinases [60]. MYC and MYBL2 play a role in cell cycle progression and act as transcription factors. MYC is also associated with apoptosis, cellular transformation, cell growth, proliferation, differentiation, and a variety of hematopoietic tumors, leukemias and lymphomas [61, 62, 63], and was part of the original proliferation signature [6]. MYBL2 has been shown to play a role in the G1/S transition [64] and proliferation [65] and is known to be regulated by CCND1 [66, 67]. CDK4 and CDK6 are important regulators of cell cycle transition from G1 to S, phosphorylate, and thus regulate the activity of tumor suppressor protein Rb [68].

Interaction partners of CCND1 are also significantly differently expressed (Figure 7): CCND1 and CDK4 are assumed to be involved in cell cycle progression of MCL, MYC is suspected of increasing MCL's proliferation rate. FOS, JUN and MYBL2 are partly known to play a role in cancer, but not explicitly in MCL. FOS ("v-fos FBJ murine osteosarcoma viral oncogene homolog") and JUN ("jun oncogene") are weakly downregulated in "b". Other interaction partners such as MYC ("V-myc myelocytomatosis viral oncogene homolog (avian)"), MYBL2 ("V-myb myeloblastosis viral oncogene homolog (avian)-like 2"), CDK4 ("Cyclin-dependent kinase 4") and CDK6 show higher gene expression values in bad prognosis patients below the median of survival.

Moreover, there are some genes with similar significance and expression difference, associated with other functions (Table 3). Most of them are associated with DNA metabolism. Three of them, "suppressor of cytokine signaling 1" (SOCS1), "tubulin, alpha 1b" (TUBA1B), and "CCAAT/enhancer binding protein (C/EBP), beta" (CEBPB) are mentioned here. CEBPB, is a transcription factor. It plays an important role in immune and inflammatory responses [42]. Additionally it can stimulate the expression of the collagen type I gene. TUBA1B encodes for an important part of the microtubules. SOCS1 is a member of cytokine-inducible inhibitors of signaling [43] and inhibits protein kinase activity.

**Table 3:** Genes separating good (s) and bad (b) prognosis not associated with cell cycle and proliferation association

**EnsemblID**	**Gene**	**p-value**	**Fold change**
ENSG00000185338	SOCS1	2.3029981E-10	1.0293059
ENSG00000123416	TBAK_HUMAN	6.1972505E-10	1.0070857
ENSG00000172216	CEBPB	7.545418E-10	0.7460686

### CGH data reveals new genes implicated in MCL outcome

**Figure 8:**
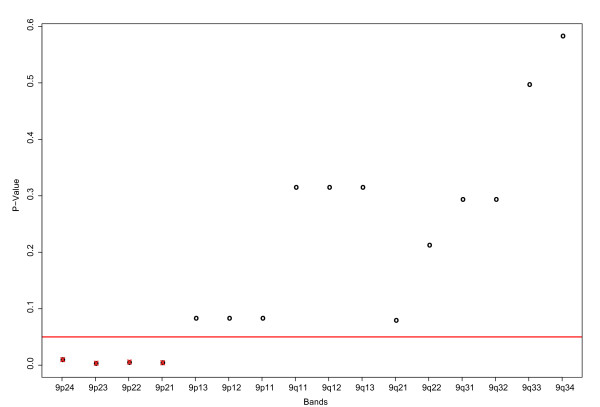
**P-values of the Wilcoxon test for the bands of chromosome 9**. This figure plots the bands of chromosome 9 on the x-axis against the p-values of the Wilcoxon test (y-axis), which tested each band between the two groups "s" and "b". The p-values of the first four bands 9p24, 9p23, 9p22, 9p21 are very small, compared to the remaining ones. This affirms the proposed subgroups "s" and "b" and indicates that the first four bands have a relation to this classification.

We applied the Wilcoxon rank sum test on the CGH data and compared the patients with good "s" and bad prognosis "b" (over and below median of survival). The null hypothesis corresponds to no differences between the two entities. The resulting p-values for every band of chromosome 9 are compared in Figure 8. They show strongly the significance of the first four bands 9p24, 9p23, 9p22 and 9p21. On these bands are MCL related genes such as "cyclin-dependent kinase inhibitor 2B" (CDKN2B) and "cyclin-dependent kinase inhibitor 2A" (CDKN2A). TP53 mutations are associated with the blastoid variant of MCL and with a worse prognosis. The bands 9q33 and 9q34 are less significant. To visualize this result more clearly we plot the densities of the p-values [see Additional file 2]. A peak in the density indicates significant bands of the Wilcoxon test.

**Figure 9:**
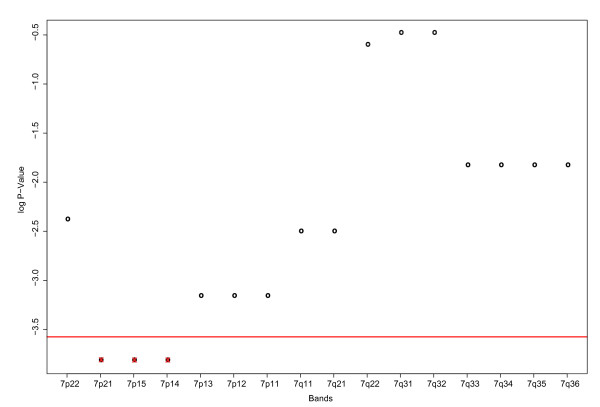
**P-values of the Wilcoxon test for the bands of chromosome 7**. The Wilcoxon test was applied to all bands of chromosome 7 over the two groups "s" and "b". The bands of chromosome 7 (x-axis) are plotted against the log p-values (y-axis). Three bands show a very low p-value: 7p21, 7p15, 7p14. As the four bands of chromosome 9, they could have a relation to the "s" – "b" classification.

The Wilcoxon rank sum test showed similar results for chromosome 7. Here, the bands 7p21, 7p15, 7p14 are potentially related to the classification of "s" and "b" patients. Now the log p-values and their densities are plotted against the bands in Figure 9, the density plot of p-values for chromosome 7 is also shown [see Additional file 3]. The explorative analyses of chromosome 7 could not show such a clear relation as in chromosome 9.

Specific gene expression differences in patients with good or bad prognosis are well supported by the CGH data of chromosome 9. We checked the location of the signature genes as we wondered if they were on chromosome 7 or 9, however this was not the case. Also the genes of the gene network in Figure 6 are located elsewhere. No result mentioned before could explain the relationship between the subgroups and the subgroup-separating CGH data of chromosome 9. We thus investigated the gene expression data of these bands. Again a moderate t-test was applied to rank genes differentially expressed between "s" and "b". The top five are listed in Table 4, e.g. the "Heat Shock 70 kDa protein 5" and a catalytic subunit of "Protein Phosphatase 6". Several of their functions implicate them to be critical in cancer development. Their genomic position revealed a quite remarkable clustering of these genes [see Additional file 4]. Three of the genes seem to be located very closely to each other. The "heat shock 70 kDa protein 5" (HSPA5), also referred to as 'immunoglobulin heavy chain-binding protein' (BiP) targets misfolded proteins for degradation, and has an anti-apoptotic property. It is induced in a wide variety of cancer cells and cancer biopsy tissues and contributes to tumor growth and confers drug resistance to cancer cells [44]. The PPP6C gene encodes for a catalytic subunit of the Ser/Thr phosphatases, the "protein phosphatase 6 catalytic subunit" [45]. The pre-B-cell leukemia transcription factor 3 (PBX3) shows extensive homology to PBX1, a human homeobox gene involved in t(1;19) translocation in acute pre-B-cell leukemia. But in contrast to PBX1 the expression of PBX3 is not restricted to particular states of differentiation or development [46]. It is also known that if HoxB8, a homeobox gene identified as a cause of leukemia, binds to the Pbx cofactors it blocks differentiation in certain cell types [47]. "Prostaglandin-endoperoxide synthase 1" (PTGS1) is the key enzyme in prostaglandin biosynthesis, and is also known to play a role in the human colon cancer [48,49]. The expression of the alternative splice variants is differentially regulated by cytokines and growth factors [50-52]. Very little is known about "quiescin Q6-like 1" (QSCN6L1), except its major role in regulating the sensitization of neuroblastoma cells for IFN-gamma-induced apoptosis [53]. A similar clear clustering as on chromosome 9 could not be detected on chromosome 7.

**Table 4:** The best "s" and "b" separating genes of chromosome 9 bands 9p24, 9p21, 9q33, and 9q34

**Gene**	**Start bp**.	**End bp**.	**Fold change**	**p-value**	**Official full name**
HSPA5	127036953	127043430	0.4364743	0.03039	Heat shock 70 kDa protein 5
PPP6C	126948673	126991918	0.2798860	0.03385	Protein phosphatase 6, catalytic subunit
PBX3	127548372	127769477	0.3976210	0.03385	Pre-B-cell leukemia homeobox 3
PTGS1	124173050	124197803	0.4124149	0.03927	Prostaglandin-endoperoxide synthase 1
QSCN6L1	138240395	138277470	-0.3886557	0.03927	Quiescin Q6 sulfhydryl oxidase 2

A moderate t-test revealed the following ones as the genes with the highest significance. Although the significance is weak, it is quite remarkable that these genes here show a distinct clustering on the basis of genomic positions [see Additional file 4].

## Discussion

Several different marker genes and events have been proposed for MCL, e.g the translocation t(11;14)(q13;q32) [1], immunohistochemically [54] and Repp86 proteins as a proliferation markers [55] and increased levels of cyclin D1.

The present study consolidates gene expression and CGH data regarding MCL subgroups with good or bad prognosis to an overall picture. These subgroups are indicated and confirmed by exploratory analyses. This picture shows as yet unknown relations and differences between patients from these groups.

Correspondence analysis is an unsupervised tool to project high dimensional data into lower dimensional subspaces. Surprisingly, its second component separates well the shorter and longer living patients according to the median of survival. This result is in close agreement with the median of the outcome predictor score derived by the proliferation signature [6] as a discriminator.

A new predictor of survival with similar predictive power as the proliferation signature of 20 genes [6] was developed requiring gene expression values of only seven genes. With the key genes CDC20, HPRT1 and CDC2 the seven-gene-predictor matches with three genes from the 20 genes proliferation signature. Moreover, the four genes CENPE, BIRC5, ASPM and IGF2BP3 add to its predictive power and are associated with chromosome movement, inhibition of apoptosis and tumors. It was shown that a four gene predictor (CDC2, ASPM, tubulin-alpha, CENP-F) [6] is also able to predict length of survival with high statistical significance. Besides the fact, that the proliferation signature is more efficient and powerful than the four gene model, our model meets extensive re-annotation of the genes through the clone IDs.

These CGH data support the association of alterations in chromosomal regions and outcome of MCL patients.

Gene expression analysis comparing long and short surviving patients delivered cell cycle related genes and their protein-protein interactions. A dense interaction network differently regulated in good or bad prognosis includes CDC2 and interaction partners for cell cycle control and proliferation (CCND1, CDK4, MYC and E2F1; CDC25, WEE1, AURKB, AURKA, BUB1, PCNA, FOS, JUN and MYBL2). However, we identified furthermore non proliferation genes differentially implicated in MCL prognosis such as SOCS1 and CEBPB.

The Wilcoxon rank sum test revealed relations between the bands 9p24, 9p23, 9p22 and 9p21 and the difference between the longer and shorter living patients. Investigation of those bands regarding most significant differentially expressed genes revealed a cluster of genes with properties such as "differentiation blocking", "anti apoptotic" and "apoptosis inducing". Supporting our finding, the band 9p21 was suggested be implicated in MCL patient outcome [56]. Some bands of chromsome 7 identified further expression differences somewhat weaker associated with the outcome. As the annotation and properties of embedded genes are not completely known, further data are required to better explain the relation between gene functions and survival. CGH data may improve the power of gene expression based predictors [57]. Besides others, the band 9p21 was associated with a poor clinical outcome, which affirms our finding.

Our study extends these CGH results in two ways: (i) exploratory analysis shows here for the first time, that in fact CGH data alone can predict prognosis in MCL, (ii) CGH data point here directly to several genes regulated differently in good or bad prognosis patients.

## Conclusion

After careful re-annotation of involved genes we found two subgroups of MCL patients which were found and supported by exploratory analysis of gene expression values and CGH data, network analysis and literature mining. We obtained an improved classification of MCL regarding prognosis. Differentially expressed genes formed a tight protein interaction network of kinases. A seven gene predictor appeared as an easy to measure prognosis indicator for clinical use. The Wilcoxon rank sum test as well as PCA was applied successfully to a CGH data set in this study. Both identify bands on chromosome 9. Following the indicated bands, we found differentially expressed MCL related genes.

## Competing interests

The author(s) declare that they have no competing interests.

## Authors' contributions

SB carried out the essential technical work for the study including data validation, calculations, statistical analysis and result figures and for Ms writing. JCE aided in these tasks including Ms. writing as well as with her expertise in analyzing gene expression data. SP as well as MW provided databank support and results. JS supervised databank support and results and added own observations. AR and HKMH provided the patient data as well as pathology expert advice during the analysis of the data and participated in the critical discussion of the results. TM supervised statistical analysis and gave expert advice including important methodological contributions. TD led and guided the study, gave supervision, led the Ms writing, and analyzed the different data and results. All authors participated in the writing of the Ms and approved the final version of the Ms.

## Pre-publication history

The pre-publication history for this paper can be accessed here:



## Supplementary Material

Additional file 5 **Gene expression ratios used in this study**. The text file contains all the data (Patients, Ensembl.ID etc.) used for the study after normalization. For the raw intensities please refer to the GEO accession number. Click here for file

Additional file 6 **Different prognosis assigned to patients**. The text file contains how different prognosis can be assigned to patients (over/below median of survival). Please refer to the paper for detailed explanation. Click here for file

Additional file 1 **Correspondence analysis of chromosome 9 over the "s" and "b" group**. The first order factor axis separates almost completely these two groups. It is also obvious that the first four bands 9p24, 9p23, 9p22, 9p21 attract most of all b-patients. This leads to the assumption, that these four bands are responsible for the difference of the longer living "s" and the shorter living "b" patients. The second order factor axis separates at first glance strongly the last two bands 9q33, 9q34 from the rest. Click here for file

Additional file 2 **Density plot of p-values of the Wilcoxon test for the bands of chromosome 9**. The p-values of Wilcoxon test for the bands (x-axis) of chromosome 9 over the subgroups "s" and "b" are represented in their relative frequencies (y-axis). The peak of the first bands indicates that signal of the test ranges from p-value 0 to 0.1. The p-values of the first four bands 9p24, 9p23, 9p22, 9p21 vary between these limits. This affirms the proposed subgroups "s" and "b" and indicates that the first four bands have a relation to this classification. Click here for file

Additional file 3 **Density plot of p-values of the Wilcoxon test for the bands of chromosome 7**. The p-values from the Wilcoxon test applied on the bands of chromosome 7 are plotted against their relative frequencies. A peak occurs between the limits of 0 and 0.1. The p-values of some bands vary between these limits. These bands are the significant signal of the performed test, affirm the proposed subgroups "s" and "b" and could have a relation to this classification. Click here for file

Additional file 4 **Plotted base pair positions of genes on Chromosome 9**. Here all genes, which are located on the bands 9p24, 9p21, 9q33, and 9q34 of chromosome 9 are sorted and plotted according to their starting genomic position. The positions are plotted on the y axis. The x-axis represents the genes. A moderate t-test revealed the best "s" and "b" separating genes in our dataset in these bands. Their starting points are drawn in red. Remarkably three are close to each other. Click here for file
