# Supplementary figures and images for: Explorative data analysis of MCL reveals gene expression networks implicated in survival and prognosis supported by explorative CGH analysis
Source: BMC Cancer. 2008 Apr 16;8:106. doi: 10.1186/1471-2407-8-106 (PMC2442114; doi:10.1186/1471-2407-8-106)

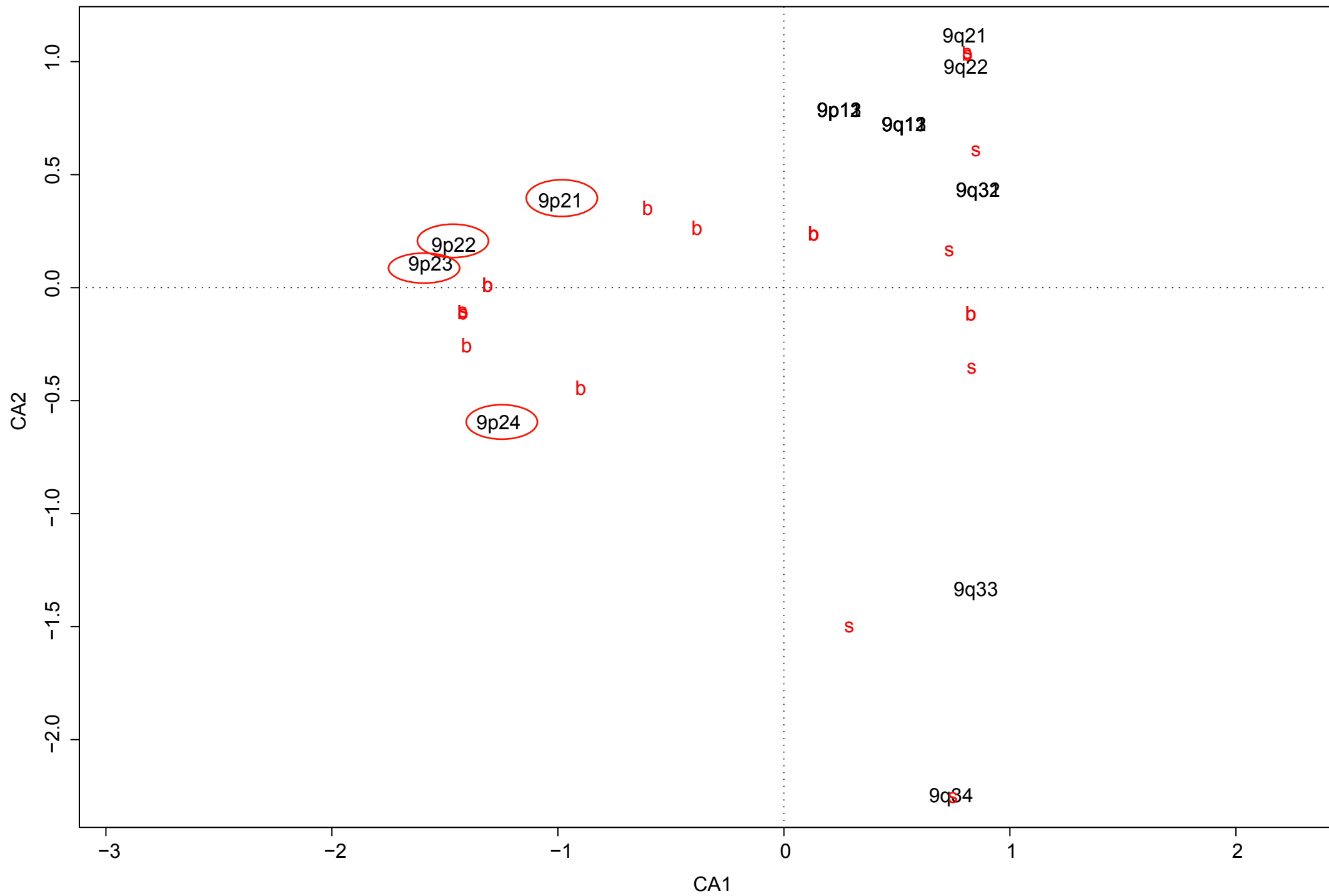

Supplement: Additional file 1 — Correspondence analysis of chromosome 9 over the "s" and "b" group. The first order factor axis separates almost completely these two groups. It is also obvious that the first four bands 9p24, 9p23, 9p22, 9p21 attract most of all b-patients. This leads to the assumption, that these four bands are responsible for the difference of the longer living "s" and the shorter living "b" patients. The second order factor axis separates at first glance strongly the last two bands 9q33, 9q34 from the rest. [file 1471-2407-8-106-S1.pdf]

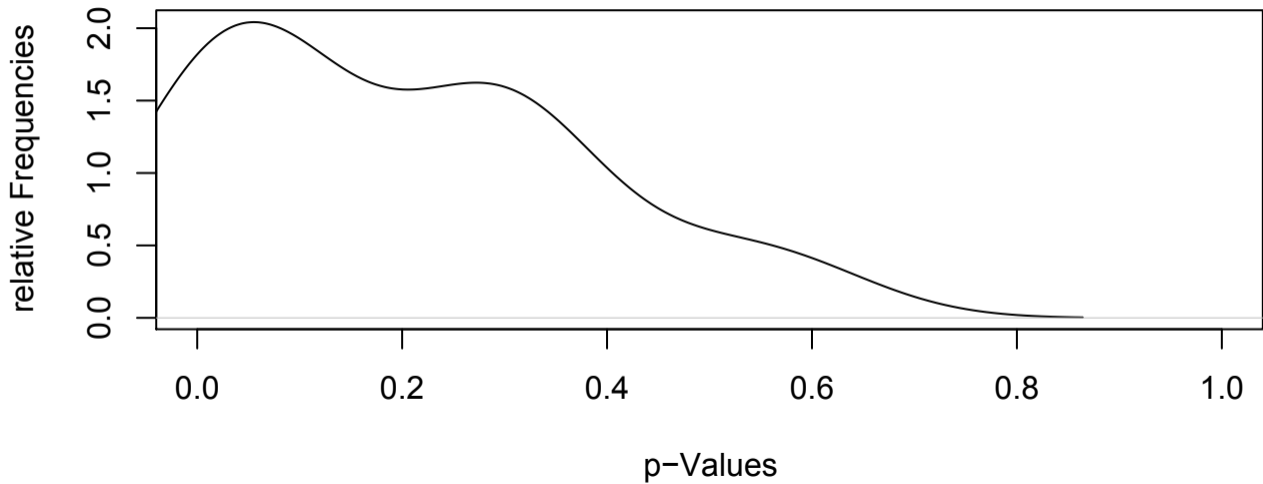

Supplement: Additional file 2 — Density plot of p-values of the Wilcoxon test for the bands of chromosome 9. The p-values of Wilcoxon test for the bands (x-axis) of chromosome 9 over the subgroups "s" and "b" are represented in their relative frequencies (y-axis). The peak of the first bands indicates that signal of the test ranges from p-value 0 to 0.1. The p-values of the first four bands 9p24, 9p23, 9p22, 9p21 vary between these limits. This affirms the proposed subgroups "s" and "b" and indicates that the first four bands have a relation to this classification. [file 1471-2407-8-106-S2.pdf]

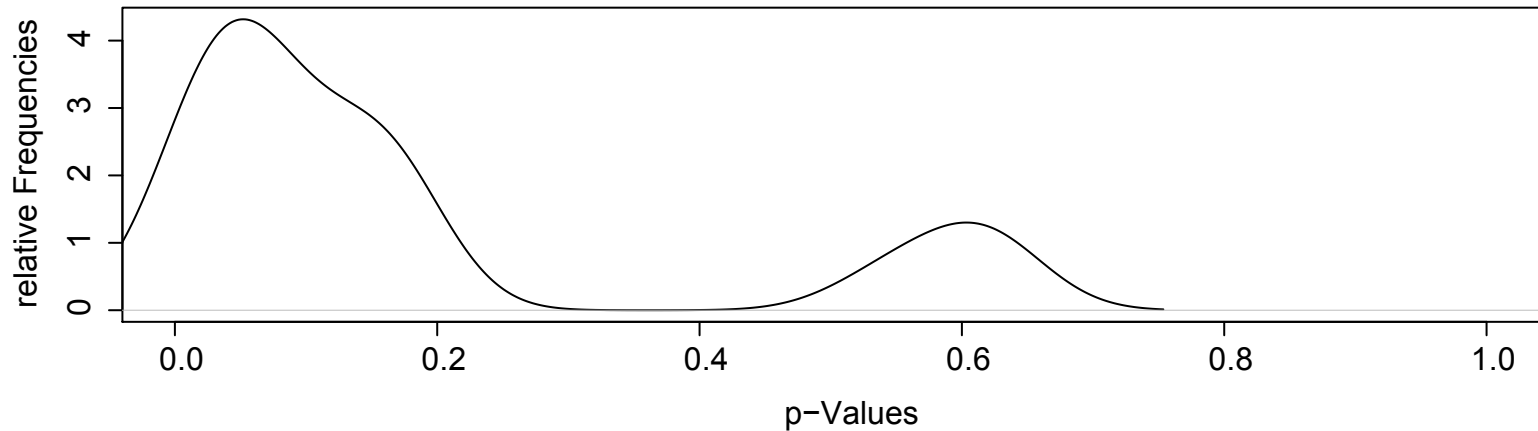

Supplement: Additional file 3 — Density plot of p-values of the Wilcoxon test for the bands of chromosome 7. The p-values from the Wilcoxon test applied on the bands of chromosome 7 are plotted against their relative frequencies. A peak occurs between the limits of 0 and 0.1. The p-values of some bands vary between these limits. These bands are the significant signal of the performed test, affirm the proposed subgroups "s" and "b" and could have a relation to this classification. [file 1471-2407-8-106-S3.pdf]

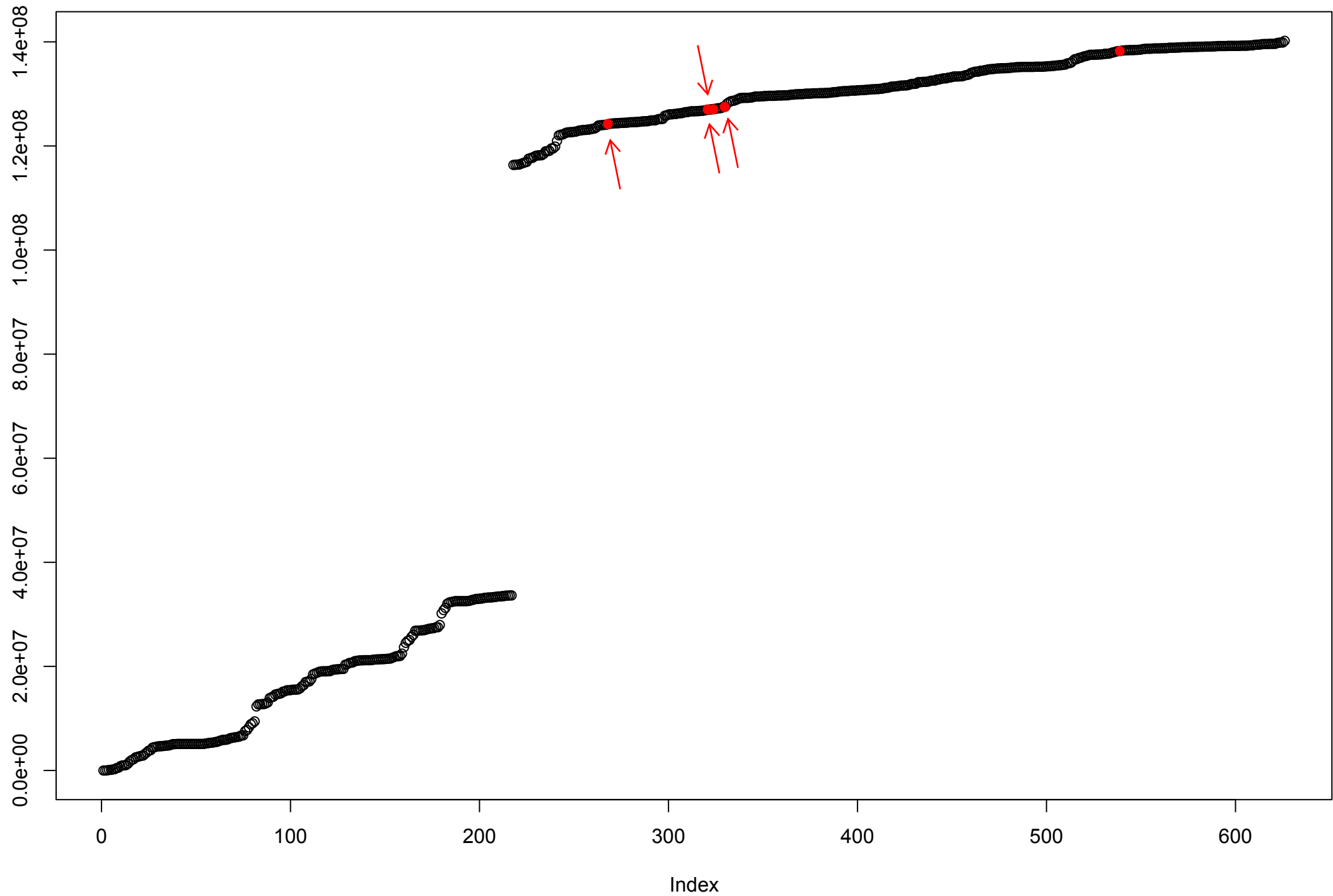

Supplement: Additional file 4 — Plotted base pair positions of genes on Chromosome 9. Here all genes, which are located on the bands 9p24, 9p21, 9q33, and 9q34 of chromosome 9 are sorted and plotted according to their starting genomic position. The positions are plotted on the y axis. The x-axis represents the genes. A moderate t-test revealed the best "s" and "b" separating genes in our dataset in these bands. Their starting points are drawn in red. Remarkably three are close to each other. [file 1471-2407-8-106-S4.pdf]
